# A novel solution for controlling hardware components of accelerators and beamlines

**DOI:** 10.1107/S1600577522002685

**Published:** 2022-04-04

**Authors:** Igor Khokhriakov, Olga Merkulova, Alexander Nozik, Petra Fromme, Victoria Mazalova

**Affiliations:** a Institute of Materials Research, Helmholtz Zentrum Geesthacht, Geesthacht, Germany; b Tango-Controls Collaboration, European Synchrotron Radiation Facility, 71 Avenue des Martyrs, 38043 Grenoble, Germany; c JetBrains Research, Kavčí Hory Office Park, Na Hřebenech II 1718/10, Praha 4 – Nusle 140 00, Czech Republic; d Center for Applied Structural Discovery, The Biodesign Institute, Arizona State University, Tempe, AZ, USA; e Centre for Free Electron Laser Science CFEL, Deutsches Elektronen-Synchrotron DESY, Notkestrasse 85, Hamburg, Germany

**Keywords:** control system, system design, reactive system, physics facility, experiment control, X-ray spectroscopy

## Abstract

A novel approach to the remote-control system for the compact multi-crystal energy-dispersive spectrometer for X-ray emission spectroscopy (XES) applications has been developed. This new approach is based on asynchronous communication between software components and on reactive design principles. In this paper, the challenges faced, their solutions, as well as the implementation and future development prospects are identified. The main motivation of this work was the development of a new holistic communication protocol that can be implemented to control various hardware components allowing both independent operation and easy integration into different SCADA systems.

## Abstract 2

A novel approach to the remote-control system for the compact multi-crystal energy-dispersive spectrometer for X-ray emission spectroscopy applications is described.

## 1. Introduction

Along with ongoing advances in the development of large-scale particle accelerators such as X-ray free-electron lasers (XFELs) and synchrotron facilities, these facilities continue to operate in a ‘high demand and limited access’ mode. The recent achievements in laser technologies open up new possibilities for the construction of compact accelerators, which will lead to an increase in the number and availability of such facilities for users delivering new technologies to universities, institutes, hospitals, *etc*. Two compact XFELs have almost been built at the Deutsches Elektronen-Synchrotron (DESY) (Kärtner *et al.*, 2016 ▸) and Arizona State University (ASU) (Graves *et al.*, 2017 ▸). A mini-synchrotron, the Munich Compact Light Source (MuCLS), operates at the Technical University of Munich (TUM) (Günther *et al.*, 2020 ▸), and other developments are underway around the world. This growth in scientific instruments has lead to a variety of implemented Supervisory Control And Data Acquisition (SCADA) systems to control the equipment. Thus, the control of technical components of accelerators is implemented with the DOOCS (Grygiel *et al.*, 1996 ▸) and TINE (Bartkiewicz & Duval, 2007 ▸) control systems, whereas the beamlines equipment is controlled with TANGO (Götz *et al.*, 2003 ▸), KARABO (Hauf *et al.*, 2019 ▸) or EPICS (Dalesio *et al.*, 1994 ▸). Such diversity of SCADA systems requires development of complicated communication protocols when synchronization and communication between different technical components is needed, especially in the facilities where multiple SCADA systems are used.

Frequently, the integration of new equipment at large-scale facilities is done by adapting the existing control software used for other devices. Such a strategy often results in replication of large databases and huge software infrastructure. In our work, we propose a different approach based on the use of modern software solutions, with the help of which one can quickly achieve similar functionality with fewer resources.

While the number of various research techniques and analytical instruments constantly grows, the idea of equipment sharing may become reasonable in the near future. Following this concept, analytical instruments for compact accelerators, when possible, should also be sufficiently compact and versatile, which includes the possibility of integration of their control system into various facilities. Good and up-to-date equipment control practice in newly developed facilities should be envisaged.

The XES spectrometer was developed for the compact XFEL beamline at DESY (the AXSIS project) and can be used for the single-shot collection of XES spectra simultaneously with X-ray diffraction/X-ray scattering experiments in a hard X-ray energy range (4–12 keV) with attosecond time resolution. One of the requirements for the XES spectrometer for the compact XFEL was its versatility, namely the possibility of installing the spectrometer at different beamlines and X-ray facilities. This requirement limits not only the weight and size of the device but also requires the spectrometer control system to be independent and easily integrated into various SCADA systems.

In our paper, we present a new solution based on asynchronous communication between software components and on reactive design principles. Our approach to hardware control can be implemented for controlling and communicating any technical equipment in real time regardless of its manufacturer, scale and complexity. We show, as an example, the implementation of the developed protocol for controlling hardware components of the X-ray spectroscopy endstation of the AXSIS project.

## 2. Challenges

### 2.1. Hardware challenges

To follow the ultrafast dynamics of chemical processes achievable with a pulsed X-ray source working at a high repetition rate, the XES spectrometer for the AXSIS project is based on the von Hamos geometry (von Hamos, 1932 ▸). Due to its cylindrical shape, each analyser crystal diffracts X-rays in a specific energy range along the cylinder axis (dispersion direction) and focuses the X-rays along the base of the cylinder (focusing direction), allowing the entire XES spectrum from each individual X-ray pulse to be measured.

The principle of operation of the XES spectrometer is based on the Bragg diffraction of X-rays from the crystal lattice planes of multiple crystal-analysers. After interaction of the incoming X-ray beam with the sample, the X-ray fluorescence is diffracted by the crystal-analysers and focused into the position-sensitive 2D detector following Bragg’s law *n*λ = 2*d*sinθ, where *n* is the diffraction order, λ is the X-ray wavelength, *d* is the distance between crystal planes, and θ is the Bragg angle.

In order to provide high efficiency in the measurement of experimental data for the low-concentrated biological samples or catalysts, the XES spectrometer was built from multiple crystal analysers increasing a covered solid angle. Unlike other existing solutions (Alonso-Mori *et al.*, 2012*a* ▸,*b* ▸; Szlachetko *et al.*, 2012 ▸), we aimed to develop a highly efficient, high resolution, compact and portable device at the same time. With this objective, the XES spectrometer for the AXSIS project consists of eight (2×4 matrix) independent crystal-analysers cylindrically bent to 250 mm. Each crystal-analyser can be moved by three individual motors to perform three different tasks: (1) change the Bragg angle (and the energy range of the detected spectra); (2) change the focus of the projection of the XES spectra to the detector, or (3) position an individual XES spectrum on a different area on the detector.

### 2.2. Software challenges

#### 2.2.1. Lightweight, compact and modular

Thinking of how the hardware tasks match with the existing software solutions we face the following. Usually existing SCADA systems, which operate with hardware, are huge and have solutions for lots of problems. Most of them are of great complexity with their own ecosystems such as libraries, servers, special tools, graphical user interfaces (GUIs) *etc*., which require efforts and resources to get into, and in-house support and development from time to time. There is no possibility to use only a part of it if the task is not big and if there are not so many drawbacks to solve. We do not need special complicated features from SCADA systems.

Hence we define the following challenge: our software must be lightweight, *i.e.* has minimal number of third-party dependencies, compact and modular – meaning that all the features are ‘on-demand’ rather than ‘built-in’ the software modules. Modules in turn should be easily interchangeable, pluggable and also required only ‘on-demand’.

For instance, as we have a multi-crystal energy-dispersive spectrometer there is no need to create and maintain a dedicated database, polling system, black-box or data archivers which are usually ‘built-in’ when using SCADA systems. So most of the existing solutions were with redundant functionality for our task.

#### 2.2.2. Portable across different beamlines

The possibility of installing the spectrometer at different beamlines and X-ray facilities was another important task to solve. Incompatibility between different systems is the real case in this situation. Devices from one SCADA system cannot be easily connected to another one because they depend on different transport protocols which are used in each SCADA system [for example, TANGO uses CORBA (Henning & Vinoski, 1999 ▸), whereas DOOCS uses SUN ONC (Srinivasan, 1995 ▸)]. The same applies for visualization services. Indeed, there are some wrappers from SCADA systems which allow connections with other systems but deep knowledge of the systems is required to implement these connectors. So when one wants to bring together two devices that use different SCADA systems, the SCADA integration has to be written from scratch. This is not convenient if the device is to be used in different facilities where different SCADA solutions are operated. Our challenge is to design a common solution to cross-integrate different SCADA systems.

#### 2.2.3. Minimizing third-party dependencies and vendor lock in a device server

Interconnecting different SCADA systems and providing visualization are not the only challenges to be solved. We also want to provide a common interface and tools to aggregate new device servers, not connected to any established platform. Those stand-alone device servers have general use-cases:

(1) Providing a way to create a device server that can interact with several control systems. Currently, one needs to create a custom device server implementation for each platform in order to make the device pluggable into them. It requires a significant effort and limits the number of devices that could be used in a specific experiment.

(2) Using device servers in a stand-alone mode, without integration with the other devices and very simple read-out and visualization. Currently, most systems do not allow running devices in this mode without setting up central naming and communication services, configuration database, storage database, *etc*.

The first case is rather self-explanatory. Device manufacturers obviously want to write a device server once using a convenient tool-set instead of supporting a device server for each platform (usually in different languages).

The second use-case mostly appears in small experiments and during component testing. It does not make sense to establish a whole SCADA system for a single device, but the drivers (device servers) are already integrated in those systems and could not be used alone. The LabVIEW platform (Travis & Kring, 2007 ▸) provides some means to manage small-scale systems, but is also limited when it comes to custom hardware.

#### 2.2.4. Making asynchronous communication a first-class citizen

Most existing control systems are using synchronous (request-response) and often one-direction communication protocols when data are propagated from the lower layers to the upper ones, *e.g.* when data are propagated from the hardware to the GUIs on request (Birrell & Nelson, 1984 ▸; Götz *et al.*, 2013 ▸), also known as client polling. This means that we either receive a command execution’s result or an error, without knowing what is going on in between. Request-response models are very good in peer-to-peer communication (Schoder *et al.*, 2005 ▸) where every node talks to another one and only one at a time, but not in multiple peer connections where a node talks to multiple nodes simultaneously. Request-response-based systems are good until we want to scale up in a multi-directional communication way. For instance, in the case where we need to add more motors, devices or software components that talk to each other, the code base quickly becomes complicated with lots of if-else statements and nested loops.

**Figure 1:**
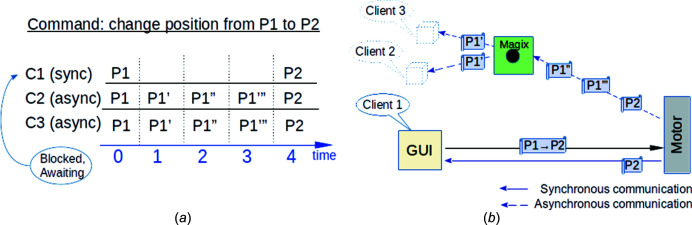
An example of the difference between synchronous and asynchronous communication. Client 1 (GUI) sends a message with a command to change the state of the motor from the current P1 position to P2 at the t0 time. After receiving the message, the motor begins to execute the command and changes its states. The motor moves through intermediate positions P1′, P1′′, P1′′′ to reach the P2 position. Via synchronous communication the Client 1 (GUI) receives response P2 at the t4 time. Whereas via asynchronous communication Client 2, Client 3 (these may be another GUI, other motors or systems), which want to receive information about intermediate motor’s states, receive these intermediate positions simultaneously: P1′ at t1 time, P1′′ at t2, P1′′′ at t3 and P2 at t4 time.

Summarizing, the synchronous communication scheme is easy to implement, but it has several disadvantages (Hintjens, 2013 ▸):

(1) Synchronicity in most cases means blocking control flow, see Fig. 1 ▸(*a*). This leads to latency and freezes in the whole system.

(2) Synchronous systems based on peer-to-peer communication do not scale well, *e.g.* dozens of peers talking to each other in a blocking way.

(3) Finally, such systems are very hard to load balance (Alakeel, 2009 ▸).

One should note that some established systems like TANGO or DOOCS now introduce an asynchronous model on a client Application Programming Interface (API) level, which is *not* an asynchronous communication for data management. In fact, an asynchronous model on a client API level uses standard synchronous RPC calls under the hood (Rauschmayer, 2018 ▸). Sometimes an event subscription model is mistakenly assumed as an asynchronous communication. Event subscription models in SCADA systems with which authors are working have critical limitations for our task and design. For example, in the event subscription model the subscription process itself is synchronous. Those limitations do not allow to fully embrace asynchronous communication. Asynchronous API and subscription have a secondary role in the existing systems, not a first-class citizen.

In order to avoid the above-mentioned drawbacks, one may use *asynchronous communication* (McCool *et al.*, 2012 ▸) and *reactive design* (Kuhn *et al.*, 2017 ▸) (see detailed description in the following chapter[Sec sec2.2.5]).

**Figure 2:**
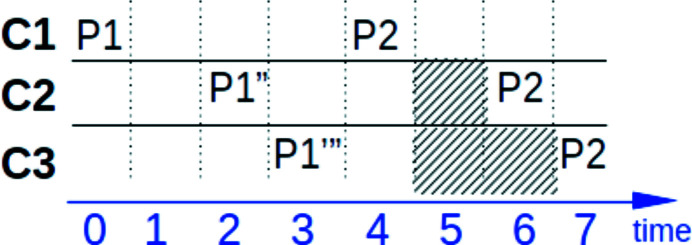
Example of synchronous communication and polling. Three clients at a different time start to ask for the motor’s position value with the time slot polling of 5. The first client C1 receives the final value of P2 at the t4 time while the second client C2 and the third C3 would receive this value only at time t6 and t7. Until the t7 time all three clients see different position values.

In our case, this means that if we send a command for the motor to change position from P1 to P2, we want to obtain intermediate positioning values of the motor (P1′, P1′′, P1′′′). An asynchronous way of communication can give us this possibility without sending constant requests from the clients’ side. Moreover, when having something like a broker [see Magix in Fig. 1 ▸(*b*)], all clients that want to get all information from the motor receive it at the same time, and this information is the same for all of them. In the case of peer-to-peer/synchronous communication, if one of the clients requests the motor’s position it may get a response with an intermediate positioning value at a time while the other client requesting for the position value will get another one. This leads to information inconsistency at some time points, see Fig. 2 ▸.

Thinking of how these tasks could be addressed, we came up with an idea of creating an open source interconnection platform – the place where information from different SCADA systems could meet and share information.

#### 2.2.5. Being reactive by design

According to our tasks, we need:

(1) To have a *responsive* solution when command execution takes time. This is solved by relying on asynchronous communication.

(2) To continue to operate in the case where one or more components fail or, in other words, to have a *resilient* system. This is provided by the way the errors are treated. Errors are messages but not exceptions that stop the process.

(3) To operate without problems and delays when a considerable number of clients (*e.g.* status monitors) are connected to the instrument – to have an *elastic* system. This is accomplished by dynamically deploying multiple broadcasters [see Magix in Fig. 1 ▸(*b*)] instances.

(4) To use messages for communication between different devices and SCADA systems.

The above statements indicate that our solution should be *responsive*, *resilient*, *elastic* and message *driven*. This means that it fits into a reactive definition or reactive manifesto (Bonér *et al.*, 2014 ▸). As we design a reactive system, message flow or message transport is to be a reactive stream which is specified in the ReactiveX Streams description (https://www.reactive-streams.org/).

One of the benefits of reactive streams is that they have a very rich ecosystem (*i.e.* libraries) also known as reactive extensions (Rx) – libraries with Rx-supported platforms (http://reactivex.io/languages.html) that can be attached to the reactive streams to extract data (information in messages) from them. These reactive extensions exist for almost all platforms and are written in most of the known software languages and provide similar functionality due to the standard reactive paradigm.

## 3. Solution

Our main goal is to provide a middleware software solution. Its main idea is to make it possible for the spectrometer and other hardware components to communicate with different control systems, GUIs and third-party software components in general. Data from one control system should be accessible for another control system in case several systems are used at once. This solution provides a generic way of communication between different SCADA systems and the instrument because there is no need to know each SCADA system to operate it. The approach, as it is, is quite widely used in web-development [especially in so-called microservice architecture (Wolff, 2016 ▸)], but is not yet fully adopted in SCADA systems. The solution is based on work done by Khokhriakov *et al.* (2014 ▸, 2017 ▸) and the ‘Troitsk nu-mass’ experiment in search of masses of active and sterile neutrinos (Abdurashitov *et al.*, 2015 ▸).

### 3.1. Message specification

**Figure 3:**
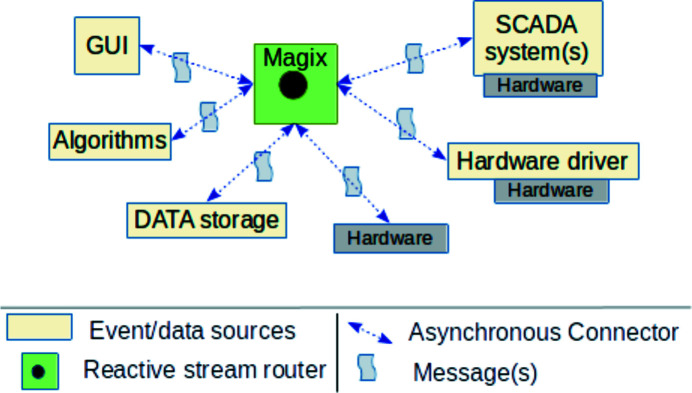
Overview of the interconnection platform. Here Magix is a core component responsible for the message flow between components. Each software component, shown as yellow boxes, deals only with incoming messages without knowing any details of the counterparty component. All communication is asynchronous.

The elementary unit of the communication process is a message. Each message is a predefined structure filled with information. This structure has been defined after analysis of SCADA systems (TANGO, TINE, DOOCS, EPICS) for more than five years and written in a Request for Comments (RFC) way (Daigle, 2007 ▸) which means that the structure can be updated if needed. Thus, for different SCADA systems the structure of the message is the same. Once a message is created it can be used/applied by clients (other SCADA systems, GUI or algorithms), see Fig. 3 ▸.

Each box in Fig. 3 ▸ (green, yellow or gray) is a solution’s component which is a stack of technologies. Depending on the functional requirements a set of components can be adjusted, thus allowing to build the required system as from Lego blocks.

### 3.2. Asynchronous communication

By design the solution implies an asynchronous way of messages’ interchange between components (see Fig. 3 ▸). When required, an asynchronous way can always be transformed into synchronous. Components may also establish peer-to-peer communication as in existing SCADA systems because the solution extends SCADA systems, not replaces them.

The core component of the solution – Magix – provides broadcasting of messages and subscribing capabilities. Messages are delivered by a transport which can be implemented by any existing transport frameworks (*e.g.* ZMQ, Kafka, *etc.*) depending on the requirements. For instance, for small instruments when the whole system is on a single host the solution can be packed into a single process. In this case, the transport may be implemented by ZMQ in-process sockets (http://api.zeromq.org/2-1:zmq-inproc). For mid to large instruments the transport may be implemented by Apache Kafka (https://kafka.apache.org/) – an event streaming platform – or D-Bus (Palmieri, 2005 ▸) or ZMQ inter-process socket (ZMQ intreprocess, http://api.zeromq.org/2-1:zmq-ipc).

### 3.3. Reactive design

Thinking about the solution, we rely on the reactive design approach. Another key feature of our solution is transport agnostic, *i.e.* irrelevance to the underlying transport implementation. The solution is implemented using the reactive stream paradigm and is not based on any existing Remote Procedure Call models (Corbin, 2011 ▸) or frameworks but based on microservices which asynchronously communicate with each other. Thus, the solution is very container friendly and can be packed into widely used Docker (Poulton, 2016 ▸) containers and orchestrated using, for example, Kubernetes (Poulton & Joglekar, 2019 ▸) to manage multiple Docker containers.

Summarizing, the main important features of the solution are:

(1) Transport agnostic.

(2) Reactive system by design.

(3) Lightweightness, compactness and modularity.

(4) Its role is to be a middleware between client applications and upstream control system(s) or third-party components.

**Figure 4:**
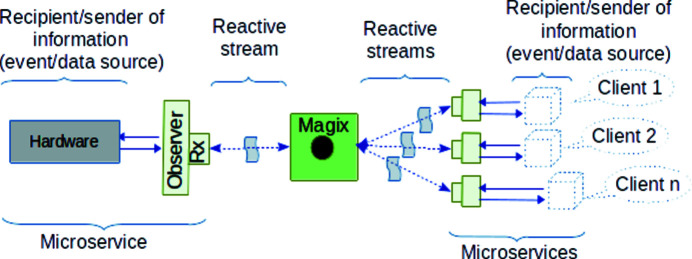
As in Fig. 3 ▸, here Magix is a core component responsible for the message flow between counterparts. Each counterpart is a software component or a microservice. Every microservice has its own lifecycle independent from others. The only thing they have in common is that they all attach to a reactive stream produced by Magix using reactive extensions (Rx) and defining observers. Observers ‘glue’ Rx and microservice’s logic.

It gives the possibility of an agile approach to software development through microservices within the reactive paradigm and makes a more convenient workflow for operating experiments, see Fig. 4 ▸.

Hence, our solution gives a variety of possibilities to connect to the streams with messages which can come from SCADA systems, drivers or any other third-party components or microservices. We deal only with a reactive stream that brings standard defined messages and there is no need to know the details of the SCADA systems, drivers or any other third-party components or microservices; we only need to understand data from messages. The messages’ structures are defined in the RFCs and the structure of the message is known in advance. Therefore, the approach allows us to use different SCADA systems as stated among the challenges.

### 3.4. Device server

Microservices are independent functional units (see green, yellow or gray boxes in Figs. 3 ▸ and 4 ▸). In order to address the challenge of reducing proprietary third-party dependencies, and especially to avoid vendor lock, we have started to design a new framework which is a combination of Rx extension (gives us the possibility to connect to the data stream and get information from messages) and Rx observer (http://reactivex.io/documentation/observable.html) (transforms message’s information in an understandable way for the recipient) which is attached to the reactive streams. The framework supports passing device property changes in and out of the device via reactive streams and Magix.

By design, the solution does not require any dedicated component that will implement the name resolution service. In other words, it does not require any particular database to be running. Small installations may benefit from it as less resources are required to set up and maintain the system. However, for large-scale installations the name resolution service can be implemented.

Since Magix compatible device servers do not have limitations on their own infrastructure (like timing services and local storage support), they could be easily built on top of existing vendor-provided solutions (*e.g.* drivers). This allows to both utilize existing libraries, not compatible with the chosen control system, and write new ones if they do not suffice.

The key details of implementation of this approach are shown in the next chapter.

## 4. Implementation

### 4.1. Hardware

**Figure 5:**
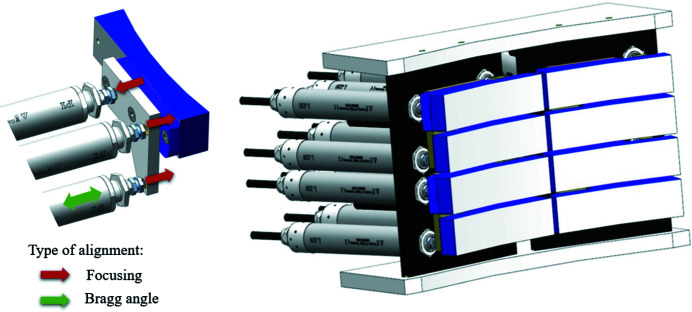
(Left panel): one crystal driven by three linear actuators. Right panel: a view of the XES spectrometer.

In order to comply with the required tasks, three main scenarios for positioning of each individual crystal of the XES spectrometer are envisaged:

(1) Positioning each crystal in the dispersion direction (changing the Bragg angle) by moving one actuator in the positive or negative direction relative to the reference position (see green arrow in Fig. 5 ▸).

(2) Changing the position of each crystal in the focusing direction by moving the three actuators sequentially or simultaneously in the positive and negative direction relative to the reference position (see red arrows in Fig. 5 ▸).

(3) Moving each crystal as a whole in a positive or negative direction relative to its original position.

All of the above-described displacements can be different for each crystal.

**Figure 6:**
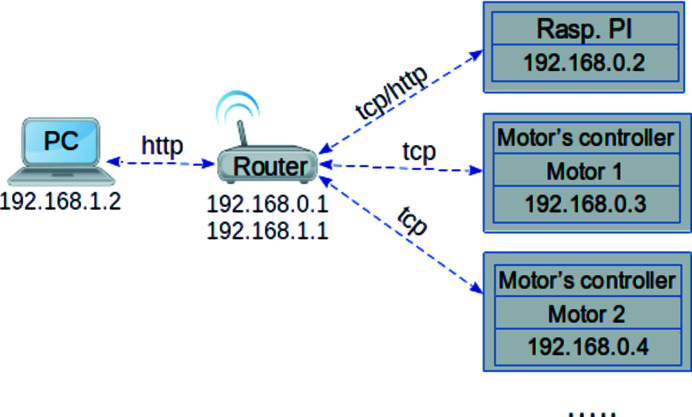
Schematics of the local network to control the XES spectrometer.

Fig. 6 ▸ shows a schematic representation of the implemented communication network for controlling the XES spectrometer. To realize these scenarios, we used 24 linear actuators driven by two controller modules (Motor 1 and Motor 2 in Fig. 6 ▸). To ensure the possibility of integrating the spectrometer into any equipment control system, a local network that includes a Raspberry PI computer and a router has been built. The Raspberry PI computer contains all the required software and libraries to control 24 linear actuators of the XES spectrometer. The router is used to provide a network communication between a remote PC and constituent elements of the spectrometer.

Any command received from the GUI on the PC is transmitted to the server deployed on the Raspberry PI. Once a command is assigned, it is redirected to the appropriate motor (or device) and its current status is returned to the user. All communications are performed asynchronously, which allows various actions to be performed in parallel.

All the software components of our system are packed in Docker containers and deployed on the Raspberry PI in Microk8s cluster (Hartwell, 2019 ▸; axsis-kube, https://github.com/Ingvord/axsis-kube).

### 4.2. Software

**Figure 7:**
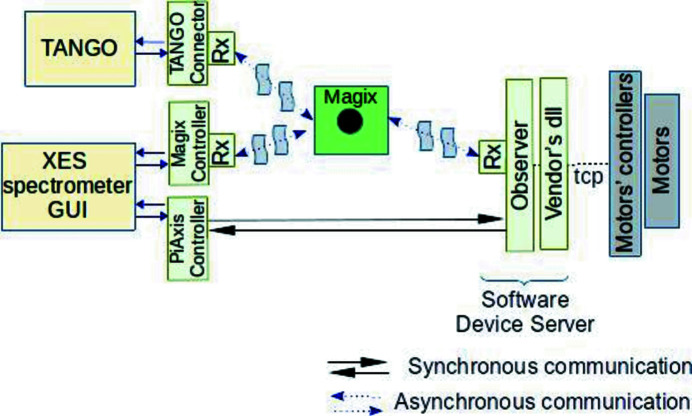
Control system implementation for the XES spectrometer. The software components may communicate both asynchronously and synchronously with other microservices via message’s flow which is used to transfer data.

In this section we give an overview of the key details of the software solution’s implementation. First, let us have a look at the GUI end of the system. As stated within the tasks, our GUI must provide a way for the user to adjust the position of each crystal as a whole by changing the Bragg angle or the focus, as well as interact with every motor independently. Obviously, it also has to be able to initiate the experiment session, connecting to the upstream components, in our case Magix and software device controllers. Information from the device server is packed into a message described in Waltz-Controls RFC-1 Message (https://github.com/waltz-controls/rfc/tree/master/1). This information can be unpacked by the control system’s connector or the GUI. The software device server is running on a dedicated host, communicating with upstream hardware devices via TCP over Ethernet or directly through USB ports. The data from devices can be visualized directly on the computer where it is acquired, see Fig. 7 ▸.

The XES spectrometer GUI is implemented on the Waltz GUI platform (https://github.com/waltz-controls/waltz) (Götz *et al.*, 2019 ▸; Khokhriakov *et al.*, 2019 ▸) as it is possible to create a user interface conforming to our needs. Moreover, it is an open source web-based solution. As a web application it does not depend on the system and can be run on Windows, Linux and mobile platforms. The XES spectrometer GUI is a typical front-end project that utilizes the ecosystem of NodeJS and JavaScript (Brown, 2014 ▸), *i.e.* dependencies management and builds are done using the npm package manager (https://www.npmjs.com/). The XES spectrometer GUI’s source code resides on GitHub (XES spectrometer GUI’s source code, https://github.com/Ingvord/axsis-xes-gui). For the graphical part we use the Webix UI library (https://webix.com) with its widgets collection (https://docs.webix.com/desktop__components.html) that provides a huge set of predefined widgets as well as a very intuitive and straightforward way of defining custom widgets and it is also distributed under an open source license.

Two points of interest within the GUI source code are asynchronous and synchronous communication with the upstream device controller. Synchronous communication with the upstream device controller is encapsulated in Pi device synchronous controller (PiAxsisController, https://github.com/Ingvord/axsis-xes-gui/blob/master/src/controllers/pi_controller.js), asynchronous communication in the MagixController component (https://github.com/Ingvord/axsis-xes-gui/blob/master/src/controllers/magix_pi_controller.js).

**Figure 8:**
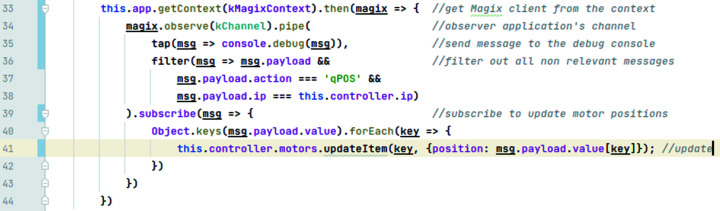
Example of a reactive update for ‘move’ action for motors.

A part of the MagixController component code example is presented in Fig. 8 ▸ to show the reactive paradigm approach. Fig. 8 ▸ shows the code snippet responsible for updating motor positions in the GUI. An asynchronous client to Magix is implemented in the reusable plugin waltz-magix-plugin (https://github.com/waltz-controls/waltz-magix-plugin). It provides an implementation of the Magix client API specification defined in the Waltz-Controls RFC-2 (https://github.com/waltz-controls/rfc/tree/master/2). Technically this plugin is not only bound to the GUI of this applications but can be used in any JavaScript application, as it is available in the NPM repository. We get the Magix client instance from the application’s context, see line 33. In lines 34–39 we observe incoming messages from the upstream device controller and update the GUI (40–42). The messages’ specification is defined in RFC-6.

The software device controller in this setup is implemented using Python 3 and Pi Python library, *i.e.* vendor library. The source code for this component resides in the XES device controllers’ source code on GitHub (https://github.com/Ingvord/axsis-xes).

To export a synchronous communication channel, we use the Flask framework for building REST services (Richardson *et al.*, 2013 ▸). This provides a very convenient way to communicate with the device controller as the HTTP eco-system, on which REST services are based, is extremely rich. For example, we can use just a plain browser to communicate with the device. In general, REST is more preferable to conventional request-response-based solutions like CORBA, see, for example, RESTful Architectural Principles by Burke (2009 ▸).

To export asynchronous communication, we developed a reusable Python client implementation for Magix (RxPython MagixClient, https://github.com/waltz-controls/magix-python-client, https://pypi.org/project/magix-client/0.4/) and attached an observer to it (see Fig. 7 ▸). The observer extracts incoming messages and delegates execution. All routines are non-blocking and performed within the async.io event loop.

An alternative implementation of the software device controller (Data acquisition framework based on DataForge, https://github.com/mipt-npm/controls.kt/tree/dev) is being developed in Kotlin using kotlin-multiplatform (https://kotlinlang.org/docs/multiplatform.html) (Nozik, 2019 ▸) technology, which has perfect instruments for implementing asynchronous communication. It allows us to share code between the Java virtual machine (JVM), browser and native targets. The key feature of the alternative implementation is bypassing vendor’s dll and direct communication with the upstream hardware devices, thus eliminating third-party dependency and fulfilling one of our challenges. The project is developed in a JetBrains Research fellowship and currently features a library for creating asynchronous device servers of different complexity as well as communication plugins that follow the Magix specification for REST/SSE, ZMQ and RSocket transport layers.

As for the Magix component, in addition to the Kotlin implementation, we developed an experimental reusable implementation based on HTTP2.0 and SSE written in Java (source code is on Magix component’s GitHub page, Source code for HTTP2.0 and SSE written in Java, https://github.com/waltz-controls/magix-war-plugin). Following RFC-2, it implements two methods – ‘subscribe’ and ‘broadcast’ – as REST endpoints.

The implementation of the solution can be considered as a reactive system. The system has to fulfill certain requirements (Bonér *et al.*, 2014 ▸) to be reactive: *message driven*, *elastic*, *resilient* and *responsive*. The implementation is naturally *message driven*. Synchronous HTTP communication is, in fact, message driven due to the asynchronous nature of JavaScript (https://developer.mozilla.org/en-US/docs/Web/JavaScript/Reference/Global_Objects/Promise) (ZeoLearn, 2018 ▸). It is *elastic* as it is easy to imagine horizontal scalability by increasing the number of Magix instances and the balance load between them. The natural limitation for scalability is the upstream hardware devices as we have a fixed number of motors. However, it is easy to leverage the load applying back-pressure strategies (Reactive Manifesto Glossary, https://www.reactivemanifesto.org/glossary#Back-Pressure) (Phelps, 2019 ▸) provided by Reactive extensions. *Resilience* is achieved by early failure interception in the software components and emitting an ‘error’ message. Finally, it is *responsive* as there are no blocking calls in the communication channels.

An important feature of our implementation is that it does not require the setup – the only thing one needs is to plug the router into the on-premise network and access the GUI using a browser. Naturally, one can switch to a different computer without stopping the measurements and restarting the acquisition.

As a proof of concept of cross integration with Tango Controls we see a virtual Tango database (virtual-tango-host, https://github.com/IK-Company/virtual-tango-host) and a dynamic Tango server that utilize dynamic attributes and commands (Götz *et al.*, 2015 ▸). The idea is to have a Tango Controls API as a front-end to our system while the back-end is connected to Magix. Hence, native Tango tools and clients can be used directly with our system. Integration with DOOCS can be implemented in a similar way, *e.g.* doocs-json (https://github.com/mipt-npm/doocs-json). Basically, integration with any third-party software component, which can be, for instance, a SCADA system or an archiving or a GUI, is done by defining corresponding messages’ specification and implementing a simple micro-service that translates those messages into upstream API calls. The deep overview of this integration approach is the subject of a separate article.

## 5. Conclusions and perspectives

In this paper, we have presented tasks, challenges, solutions and implementation of the XES spectrometer and its control system. Specifically, our hardware solution has to be compact and versatile, also in terms of software, and can be easily integrated into existing instruments.

In terms of hardware tasks for the short-working-distance multi-crystal von Hamos spectrometer, we ended up with a non-commercial customized solution that meets the requirement of the AXSIS project at DESY.

The software control system must provide the possibility to understand what is happening with the hardware while executing the commands. This presents us with the following challenges: the software solution must be responsive, resilient, elastic and message-driven.

To meet the software challenges, we developed a system based on the reactive paradigm whereas existing solutions are typically RPC systems and are not suitable as they do not provide asynchronous communication and also typically are very complex and complicated for our compact device. They also demand the creation and maintenance of a database, which we would like to avoid.

Our solution is transport agnostic and we foresee components implemented in other languages or technology stacks, *e.g.* Python and Kotlin-Native. Moreover, using Magix we can extend the capabilities of any existing SCADA, distributed control system (DCS) solution allowing easy integration of new protocols, smooth migration to new protocols, or event technology stacks.

The resulting implementation is open source. Most of the components are reusable and reside under the Waltz-Controls organization on GitHub (https://github.com/waltz-controls). Magix-related components are aggregated in the Piazza project (https://github.com/waltz-controls/piazza). The software device server is implemented in both Kotlin and Python languages. This implementation can be easily scaled to integrate more hardware and software components.

In this paper, we intentionally omit the part of the system responsible for integration with, for example, Tango Controls, and present a general overview of the challenges and the solution. Being short, integration with Tango Controls is implemented by providing a virtual Tango database (virtual-tango-host) and a dynamic Tango server. There is also some work done with the integration with DOOCS (doocs-json). A common solution to cross-integrate different SCADA systems is foreseen within the Piazza project.

At first, it may seem that the solution is over-engineered, especially in terms of the variety of technology stacks used; this was done on purpose just to demonstrate that the solution is very flexible, lightweight, compact and modular. Almost any technology stack can be used to implement its components; the only dependency is a reactive extension for a given platform.

The advantage of our solution is that it is based on messages defined in RFCs. These messages can be implemented in any language. This is a different implementation method compared with other existing control systems known to the authors of this article.

The solution has been adopted for a slow control system developed for the BabyAIXO project; see, for example, Section 8.3 of Abeln *et al.* (2021 ▸).

The XES spectrometer and its control system have been successfully tested during a research and development (R&D) beam time at Pohang Accelerator Laboratory X-ray Free Electron Laser (PAL-XFEL).

## References

[bb1] Abdurashitov, D., Belesev, A., Berlev, A., Chernov, V., Geraskin, E., Golubev, A., Koroteev, G., Likhovid, N., Lokhov, A., Markin, A., Nozik, A., Parfenov, V. I., Skasyrskaya, A. K., Pantuev, V. S., Titov, N., Tkachev, I., Tkachov, F. & Zadorozhny, S. (2015). *J. Instrum.* **10**, T10005.

[bb2] Abeln, A. K., Altenmüller, S., Arguedas Cuendis, E., Armengaud, D., Attié, S., Aune, S., Basso, L., Bergé, B., Biasuzzi, P. T. C., Borges De Sousa, P., Brun, N., Bykovskiy, D., Calvet, J. M., Carmona, J. F., Castel, S., Cebrián, V., Chernov, F. E., Christensen, M. M., Civitani, C., Cogollos, T., Dafní, A., Derbin, K., Desch, D., Díez, M., Dinter, B., Döbrich, I., Drachnev, A., Dudarev, L., Dumoulin, D. D. M., Ferreira, E., Ferrer-Ribas, I., Fleck, J., Galán, D., Gascón, L., Gastaldo, M., Giannotti, Y., Giomataris, A., Giuliani, S., Gninenko, J., Golm, N., Golubev, L., Hagge, J., Hahn, C. J., Hailey, D., Hengstler, P. L., Henriksen, T., Houdy, R., Iglesias-Marzoa, F. J., Iguaz, I. G., Irastorza, C., Iñiguez, K., Jakovcic, J., Kaminski, B., Kanoute, S., Karstensen, L., Kravchuk, B., Lakic, T., Lasserre, P., Laurent, O., Limousin, A., Lindner, M., Loidl, I., Lomskaya, G., López-Alegre, B., Lubsandorzhiev, K., Ludwig, G., Luzón, C., Malbrunot, C., Margalejo, A., Marin-Franch, S., Marnieros, F., Marutzky, J., Mauricio, Y., Menesguen, M., Mentink, S., Mertens, F., Mescia, J., Miralda-Escudé, H., Mirallas, J. P., Mols, V., Muratova, X. F., Navick, C., Nones, A., Notari, A., Nozik, L., Obis, C., Oriol, F., Orsini, A., Ortiz de Solórzano, S., Oster, H. P., Pais Da Silva, V., Pantuev, T., Papaevangelou, G., Pareschi, K., Perez, O., Pérez, E., Picatoste, M. J., Pivovaroff, D. V., Poda, J., Redondo, A., Ringwald, M., Rodrigues, F., Rueda-Teruel, S., Rueda-Teruel, E., Ruiz-Choliz, J., Ruz, E. O., Saemann, J., Salvado, T., Schiffer, S., Schmidt, U., Schneekloth, M., Schott, L., Segui, F., ten Tavecchio, H. H. J., Kate, I., Tkachev, S., Troitsky, D., Unger, E., Unzhakov, N., Ushakov, J. K., Vogel, D., Voronin, A., Weltman, U., Werthenbach, W., Wuensch, A. & Yanes-Díaz (2021). *arXiv*:2010.12076 [physics.ins-det].

[bb3] Alakeel, A. (2009). *Intl J. Comp. Sci. Network Security*, **10**, 153–156.

[bb4] Alonso-Mori, R., Kern, J., Gildea, R. J., Sokaras, D., Weng, T.-C., Lassalle-Kaiser, B., Tran, R., Hattne, J., Laksmono, H., Hellmich, J., Glöckner, C., Echols, N., Sierra, R. G., Schafer, D. W., Sellberg, J., Kenney, C., Herbst, R., Pines, J., Hart, P., Herrmann, S., Grosse-Kunstleve, R. W., Latimer, M. J., Fry, A. R., Messerschmidt, M. M., Miahnahri, A., Seibert, M. M., Zwart, P. H., White, W. E., Adams, P. D., Bogan, M. J., Boutet, S., Williams, G. J., Zouni, A., Messinger, J., Glatzel, P., Sauter, N. K., Yachandra, V. K., Yano, J. & Bergmann, U. (2012*a*). *Proc. Natl Acad. Sci. USA*, **109**, 19103–19107.

[bb5] Alonso-Mori, R., Kern, J., Sokaras, D., Weng, T.-C., Nordlund, D., Tran, R., Montanez, P., Delor, J., Yachandra, V. K., Yano, J. & Bergmann, U. (2012*b*). *Rev. Sci. Instrum.* **83**, 073114. 10.1063/1.4737630 PMC3422323 22852678

[bb9] Bartkiewicz, P. & Duval, P. (2007). *Meas. Sci. Technol.* **18**, 2379–2386.

[bb10] Birrell, A. D. & Nelson, B. J. (1984). *ACM Trans. Comput. Syst.* **2**, 39–59.

[bb11] Bonér, J., Farley, D., Kuhn, R. & Thompson, M. (2014). *The Reactive Manifesto*, https://www.reactivemanifesto.org/.

[bb12] Brown, E. (2014). *Web Development with Node and Express: Leveraging the JavaScript Stack.* O’Reilly Media.

[bb13] Burke, B. (2009). *RESTful Java with JAX-RS 2.0: Designing and Developing Distributed Web Services.* O’Reilly Media.

[bb14] Corbin, J. R. (2011). *The Art of Distributed Applications: Programming Techniques for Remote Procedure Calls.* Springer.

[bb15] Daigle, L. (2007). *The RFC Series and RFC Editor*, https://doi.org/10.17487/RFC4844.

[bb16] Dalesio, L., Hill, J., Kraimer, M., Lewis, S., Murray, D., Hunt, S., Watson, W., Clausen, M. & Dalesio, J. (1994). *Nucl. Instrum. Methods Phys. Res. A*, **352**, 179–184.

[bb19] Götz, A., Bourtembourg, R., Chaize, J.-M., Verdier, P., Coutinho, T., Moldes, J., Pivetta, L., Khokhriakov, I., Merkulova, O., Gara, S., Goryl, P., Liszcz, M., Joubert, A., Abeille, G., Mant, G., Braun, T., Hardion, V. & Bartolini, M. (2019). *Proceedings of the 17th International Conference on Accelerator and Large Experimental Physics Control Systems (ICALEPCS2019)*, 5–11 October 2019, New York, NY, USA, pp. 1234–1239. WEPHA058.

[bb20] Götz, A., Chaize, J.-M., Taurel, E., Vierdier, P., Pons, J.-L., Coutinho, T., Poncet, F., Bourtemburg, R., Abeille, G., Fulop, L. J., Gernaianu, M. O., Knapic, C. & Khokhriakov, I. (2015). *Proceedings of the 2015 International Conference on Accelerator and Large Experimental Physics Control Systems (ICALEPCS2015)*, 17–23 October 2015, Melbourne, Australia, pp. 585–588. WEA3O01.

[bb21] Götz, A., Taurel, E., Pons, J., Verdier, P., Chaize, J., Meyer, J., Poncet, F., Heunen, G., Götz, E., Buteau, A., Leclercq, N. & Ounsy, M. (2003). *Proceedings of the 2003 International Conference on Accelerator and Large Experimental Physics Control Systems (ICALEPCS2003)*, 13–17 October 2003, Gyeongju, Korea, pp. 220–222. MP705.

[bb22] Götz, A., Taurel, E., Verdier, P. & Abeille, G. (2013). *Proceedings of the 14th International Conference on Accelerator and Large Experimental Physics Control Systems (ICALEPCS2013)*, 6–11 October 2013, San Francisco, CA, USA, pp. 964–968. TUCOCB07.

[bb23] Graves, W., Chen, J., Fromme, P., Holl, M., Kirian, R., Malin, L., Schmidt, K., Spence, J., Underhill, M., Weierstall, U., Zatsepin, N., Zhang, C., Brown, D., Hong, K., Moncton, D., Nanni, A. & Limborg-Deprey, C. (2017). *Proceedings of the 38th International Free Electron Laser Conference (FEL2017)*, 20–25 August 2017, Santa Fe, NM, USA, pp. 225–228. TUB03.

[bb24] Grygiel, G., Hensler, O. & Rehlich, K. (1996). *Proceedings of the 1st International Workshop on Emerging Technologies and Scientific Facilities Controls (PCaPAC96)*. DESY, Hamburg, Germany.

[bb25] Günther, B., Gradl, R., Jud, C., Eggl, E., Huang, J., Kulpe, S., Achterhold, K., Gleich, B., Dierolf, M. & Pfeiffer, F. (2020). *J. Synchrotron Rad.* **27**, 1395–1414. 10.1107/S1600577520008309 PMC7467334 32876618

[bb26] Hamos, L. von (1932). *Naturwissenschaften*, **20**, 705.

[bb27] Hartwell, C. (2019). *Single-Node Kubernetes on Raspberry PI with MicroK8s and Ubuntu*, https://ubuntu.com/blog/single-node-kubernetes-on-raspberry-pi-microk8s-ubuntu.

[bb28] Hauf, S., Heisen, B., Aplin, S., Beg, M., Bergemann, M., Bondar, V., Boukhelef, D., Danilevsky, C., Ehsan, W., Essenov, S., Fabbri, R., Flucke, G., Fulla Marsa, D., Göries, D., Giovanetti, G., Hickin, D., Jarosiewicz, T., Kamil, E., Khakhulin, D., Klimovskaia, A., Kluyver, T., Kirienko, Y., Kuhn, M., Maia, L., Mamchyk, D., Mariani, V., Mekinda, L., Michelat, T., Münnich, A., Padee, A., Parenti, A., Santos, H., Silenzi, A., Teichmann, M., Weger, K., Wiggins, J., Wrona, K., Xu, C., Youngman, C., Zhu, J., Fangohr, H. & Brockhauser, S. (2019). *J. Synchrotron Rad.* **26**, 1448–1461. 10.1107/S1600577519006696 31490132

[bb29] Henning, M. & Vinoski, S. (1999). *Advanced CORBA Programming with C++.* Addison Wesley.

[bb30] Hintjens, P. (2013). *ZeroMQ.* O’Reilly Media.

[bb32] Kärtner, F. X., Ahr, F., Calendron, A.-L., Çankaya, H., Carbajo, S., Chang, G., Cirmi, G., Dörner, K., Dorda, U., Fallahi, A., Hartin, A., Hemmer, M., Hobbs, R., Hua, Y., Huang, W. R., Letrun, R., Matlis, N., Mazalova, V., Mücke, O. D., Nanni, E., Putnam, W., Ravi, K., Reichert, F., Sarrou, I., Wu, X., Yahaghi, A., Ye, H., Zapata, L., Zhang, D., Zhou, C., Miller, R. J. D., Berggren, K. K., Graafsma, H., Meents, A., Assmann, R. W., Chapman, H. N. & Fromme, P. (2016). *Nucl. Instrum. Methods Phys. Res. A*, **829**, 24–29. 10.1016/j.nima.2016.02.080 PMC5502815 28706325

[bb33] Khokhriakov, I., Lottermoser, L. & Beckmann, F. (2017). *Proc. SPIE*, **10391**, 103911H.

[bb34] Khokhriakov, I., Lottermoser, L., Gehrke, R., Kracht, T., Wintersberger, E., Kopmann, A., Vogelgesang, M. & Beckmann, F. (2014). *Proc. SPIE*, **9212**, 921217.

[bb35] Khokhriakov, I., Wilde, F. & Merkulova, O. (2019). *Proceedings of the 17th International Conference on Accelerator and Large Experimental Physics Control Systems (ICALEPCS’19)*, 5–11 October 2019, New York, NY, USA, pp. 1519–1524. WESH3003.

[bb37] Kuhn, R., Hanafee, B. & Allen, J. (2017). *Reactive Design Patterns.* Manning Publications.

[bb40] McCool, M., Reinders, J. & Robison, A. (2012). *Structured Parallel Programming: Patterns for Efficient Computation.* Morgan Kaufmann Publishers.

[bb41] Nozik, A. (2019). *AIP Conf. Proc.* **2163**, 040004.

[bb43] Palmieri, J. (2005). *Get on D-BUS. Red Hat Magazine*, https://web.archive.org/web/20151023072022/http://www.redhat.com/magazine/003jan05/features/dbus/.

[bb44] Phelps, J. (2019). *Backpressure explained – the resisted flow of data through software*, https://medium.com/@jayphelps/backpressure-explained-the-flow-of-data-through-software-2350b3e77ce7.

[bb47] Poulton, N. (2016). *Docker Deep Dive: Zero to Docker in a single book.* ASIN: B01LXWQUFF.

[bb48] Poulton, N. & Joglekar, P. (2019). *The Kubernetes Book: The Fastest Way to Get Your Head Around Kubernetes.* Packt Publishing.

[bb49] Rauschmayer, A. (2018). *Exploring ES6*, ch. 25, https://exploringjs.com/es6/ch_promises.html.

[bb51] Richardson, L., Amundsen, M. & Ruby, S. (2013). *RESTful Web APIs: Services for a Changing World*, 1st ed. O’Reilly Media.

[bb55] Schoder, D., Fischbach, K. & Schmitt, C. (2005). *Core Concepts in Peer-to-Peer Networking*, pp. 1–27. Idea Group Inc, https://doi.org/10.4018/978-1-59140-429-3.ch001.

[bb56] Srinivasan, R. (1995). *Remote Procedure Call Protocol Specification Version 2*, https://www.freesoft.org/CIE/RFC/1831/index.htm.

[bb57] Szlachetko, J., Nachtegaal, M., de Boni, E., Willimann, M., Safonova, O., Sa, J., Smolentsev, G., Szlachetko, M., van Bokhoven, J. A., Dousse, J., Hoszowska, J., Kayser, Y., Jagodzinski, P., Bergamaschi, A., Schmitt, B., David, C. & Lücke, A. (2012). *Rev. Sci. Instrum.* **83**, 103105. 10.1063/1.4756691 23126749

[bb58] Travis, J. & Kring, J. (2007). *LabVIEW for everyone: graphical programming made easy and fun*, 3rd ed. Upper Saddle River, NJ: Prentice Hall.

[bb67] Wolff, E. (2016). *Microservices: Flexible Software Architecture.* Addison-Wesley Professional.

[bb70] ZeoLearn (2018). *Asynchronous Nature of JavaScript*, https://medium.com/@zeolearn/asynchronous-nature-of-javascript-d7d18a71aa35.

